# Association between peer behaviors and family environment and pre-packaged sugar-sweetened beverage consumption among primary and secondary school students in Beijing

**DOI:** 10.3389/fpubh.2025.1661141

**Published:** 2025-10-01

**Authors:** Yiran Li, Lulu Meng, Siyu Liang, Yan Zhang, Wenjia Li, Jiali Duan, Ruoxiang Cao, Jie Li, Liyu Huang

**Affiliations:** ^1^School of Public Health, Hebei Medical University, Shijiazhuang, Hebei, China; ^2^Office of Research and Teaching Administration, Beijing Center for Disease Prevention and Control, Beijing, China; ^3^Institute of Nutrition and Food Hygiene, Fengtai District Center for Disease Prevention and Control, Beijing, China; ^4^Institute of Nutrition and Food Hygiene, Beijing Center for Disease Prevention and Control, Beijing, China; ^5^School of Public Health, Capital Medical University, Beijing, China

**Keywords:** sugar-sweetened beverages, children, peer, family, school-age

## Abstract

**Background:**

Sugar-sweetened beverage (SSB) consumption among children and adolescents remains high worldwide. In China, most studies have examined either peer or family influences, but few have assessed their combined effects, particularly for pre-packaged SSBs.

**Methods:**

A cross-sectional study was conducted among 2,317 primary and secondary school students in Beijing between October and November 2024. Students and their caregivers completed paired questionnaires on pre-packaged SSB consumption and potential influencing factors. Multivariable logistic regression was used to examine associations between peer and family factors and SSB consumption, adjusting for demographic and behavioral covariates.

**Results:**

In the past week, 81.9% of students consumed at least one type of pre-packaged SSB. With the increase of age, the intake of SSBs increased in children. Fruit/vegetable beverages were the most commonly consumed beverages for primary school students, and tea beverages were the most commonly consumed beverages for secondary school students. Peer-influenced purchasing behavior (OR: 1.600,95% CI:1.318–1.941), peer-sharing behavior (OR: 1.373,95% CI:1.106–1.704), household accessibility (OR: 1.305,95% CI:1.085–1.570) and parental supportive attitudes toward SSBs (OR: 2.246,95% CI:1.691–2.981) were associated with high consumption of SSBs. Similar associations were observed for carbonated beverages, tea beverages, milk beverages, and other categories, though effect sizes varied.

**Conclusion:**

Peer behaviors and family environments substantially influence pre-packaged SSB consumption among children and adolescents in Beijing. Targeted interventions should include banning SSB sales in school canteens, introducing warning labels on high-sugar beverages, and promoting parental education to reduce home availability.

## 1 Introduction

In recent years, the high consumption rate of sugar-sweetened beverages (SSBs) has become a major global challenge for children and adolescents health. Sugar-sweetened beverages, characterized by low dietary fiber and protein, high sugar content, and high energy density, have been categorized as ultra-processed foods, contributing to the global disease burden (1). Studies have shown that long-term intake of SSBs increases the risk of obesity (2), the incidence of type 2 diabetes (3), dental caries (4), and other metabolic diseases (5) in children and adolescents. The World Health Organization (WHO) has explicitly recommended that the intake of free sugars in children and adolescents should be kept below 10% of total daily energy intake, and even recommended a further reduction to less than 5% to minimize health risks (6). However, despite interventions such as sugar taxes (7, 8), advertising restrictions (9, 10), and health education intervention (11, 12) by many governments, the consumption of SSBs among children and adolescents is still generally high (2, 13). In China, despite WHO recommendations, most provincial school nutrition guidelines do not explicitly limit SSBs, and enforcement varies across regions.

In China, economic development and consumption upgrades have greatly increased the accessibility of SSBs among children and adolescents. A study pointed out that 87.6% children consumed SSB and the median consumption of SSB was 205.4 ml/day per consumer (14). A study of 25,893 Chinese adolescents aged 13–15 years showed that the proportions of Chinese adolescents who consumed sugar-sweetened beverage ≥1 times/week was 65.02% and that high-frequency sugar-sweetened beverage consumption is associated with lower muscle strength (15). The number of deaths and disease burdens caused by high sugar-sweetened beverages intake in China has increased significantly over the past three decades (16). Existing studies have largely focused on prevalence and health outcomes (17–21), while research on social determinants—particularly the roles of peers and family—remains limited in China.

Social influences are known to be critical in shaping dietary behaviors during childhood and adolescence. According to social learning theory, children often imitate the behaviors of peers and family members, especially in shared settings such as schools and homes. A study in Northeast China explored the association between family-related factors, community environmental factors, and children's sugary beverage intake, but ignored peer factors (22). As children's earliest dietary role models and food providers, parents' behaviors, attitudes and family eating environment have a profound impact on children's beverage choices. Parents' own consumption habits of SSBs, whether they store such beverages at home, and whether they actively limit their children's intake are closely related to their children's actual intake (23). Similarly, peer norms, peer pressure, and social sharing behaviors have shown to influence SSBs consumption patterns (24).

Given the scarcity of evidence on how peer and family environments influence SSB consumption among Chinese children and adolescents, this study aimed to examine the associations between these factors and the consumption of pre-packaged SSBs among primary and secondary school students in Beijing. By identifying these social determinants, the research intends to inform targeted intervention strategies that involve families and peer groups to effectively reduce sugary beverage intake among youth.

## 2 Materials and methods

### 2.1 Study population

The cross-sectional study was conducted from October to November 2024. The students were selected using a multistage stratified cluster sampling method: (1) One urban area and one suburb of Beijing were randomly selected as project sites. (2) Two primary schools, two middle schools and two high schools were selected in total across the project site, yielding eight schools overall, including four schools with nine-year consistent education (covering both primary and middle levels). (3) All students in Grade 3, Grade 7 and Grade 10 were randomly selected from each primary school, middle school and high school.

The sample size was calculated based on the beverage consumption rate (*p* = 25%) among children and adolescents reported in the Chinese Dietary Guidelines Scientific Research Report (2021) (25). *p* = 25%, μ_α/2_ 1.96, δ = 0.15p, and the design effect (deff) = 2, the required sample size was calculated as *n* = 1,024. Considering stratification by urban and suburban areas (two strata) and an anticipated 10% invalid questionnaire and non-response rate, the final estimated sample size was 2,276 participants. Using the formula:


$$\begin{array}{l} n=\frac{{\mu^{2}}_{\alpha /2}\times p\;\left(1-p\right)}{\delta^{2}}\times deff \end{array}$$

Participants were selected based on the following criteria: (1) Voluntarily undergo this survey and sign the informed consent form; (2) No plans to move out or transfer in the future; (3) Students who can cooperate in completing the questionnaire. Exclusion criteria: (1) Not sign the informed consent form and unable to cooperate with the personnel who are unwilling to cooperate with the questionnaire; (2) Leave of absence or have recent plan to move out or transfer to another school. A total of 2,525 student–caregiver pairs were recruited. After excluding the cases with incomplete key variables, 2,317 pairs (response rate: 91.8%) were included in the final analysis.

The study was approved by the Ethics Committee of the Beijing Center for Disease Prevention and Control (BJCDC2024031). All participants and their caregivers co-signed a written informed consent form prior to participation in the study.

### 2.2 Measurements and variable definition

A self-designed questionnaire was used for the survey. Based on a review of relevant literature, the research team developed the questionnaire, which was subsequently reviewed and revised by experts before being finalized as the Sugar-Sweetened Beverage Consumption Behavior Questionnaire (86 items) and the Family Environment Questionnaire on Sugar-Sweetened Beverage Consumption Behavior (68 items). A pilot test indicated Cronbach's α coefficients of 0.81 and 0.75, respectively, demonstrating good reliability.

The questionnaire mainly covered participants' demographic characteristics, knowledge and attitudes toward sugar-sweetened beverages, consumption behaviors, and related health behaviors. The Sugar-Sweetened Beverage Consumption Behavior Questionnaire and the Family Environment Questionnaire on Sugar-Sweetened Beverage Consumption Behavior were completed independently by students and their parents, respectively. Student questionnaires were administered in schools under teacher supervision, while parent questionnaires were self-administered at home. Student and parent responses were matched one-to-one through questionnaire coding.

#### 2.2.1 Consumption of sugar-sweetened beverages

Students report the frequency and amount of SSBs consumption over the past week. The questionnaire is developed based on the BEVQ-15 (26), and a modified version of the questionnaire is used to measure children's consumption of SSBs, which includes two categories: pre-packaged beverages and freshly made beverages. Beverages are classified according to the nutritional components of each beverage and the Chinese General Standard for Beverages (GB/T 10789-2015) (27). There are 8 categories of pre-packaged beverages, including 100% fruit/vegetable juices, fruit/vegetable beverages, carbonated beverages, tea beverages, milk beverages, plant protein drinks, beverages for special uses, coffee beverages. The Cronbach α coefficient is 0.817, indicating good internal consistency.

To give students a better understanding of the types of beverages, we have listed several common examples of SSBs categories to improve interpretability: vegetable- or fruit-flavored beverages that were not 100% fruit or vegetable juice (hereafter referred to as “fruit/vegetable beverages”, e.g., Minute Maid orange juice), carbonated beverages (e.g., cola, Sprite), tea beverages (e.g., iced tea, jasmine tea), sugar-sweetened milk beverages that were not milk or yogurt (hereafter referred to as “milk beverages”, e.g., Fruity milk, Nutri-Express), plant protein beverages (e.g., soya-bean milk drink, walnut drink, almond milk drink), beverages for special uses (e.g., sports drinks, energy drinks, nutrient drinks, electrolyte drinks, such as Red Bull, Pulse) (28).

To assess the weekly consumption of beverages, the questionnaire sets “Drinking Frequency” and “Average Drinking Per Consumption”. “Drinking frequency” is set to “times/day” (drinking once or more a day is reported here), “times/week” (drinking at least once a week, but not consuming every day is reported here), “never” (not drinking in the past week is reported here). Students were asked to report the frequency and amount of each drink according to their actual situation, and the amount of drinking measured in milliliters. Finally, the frequency of each drink is unified as “times/week”. SSBs are considered “consumed” when the frequency of consumption of SSBs in any of the above categories is not “never”.

#### 2.2.2 Health-related behaviors

For the purpose of this study, health-related behaviors refer to average daily water intake (<1,000 ml, 1,000–1,500 ml, >1,500 ml), outdoors activity time (< 60 min/day, ≥60 min/day), and weekday/weekend sleep duration. According to the Notice on Further Strengthening the Sleep Management of Primary and Secondary School Students issued by the Ministry of Education of China in 2021, primary school students should sleep for 10 h a day, junior high school students should sleep for 9 h a day, and high school students should sleep for 8 h a day.

#### 2.2.3 Peer-related factors

In this study, students were asked “Do you purchase sugary drinks because your friends or classmates do“ and “Do you share sugar drinks with your friends or classmates.” Answer is dichotomous variable: yes or no.

#### 2.2.4 Family-related factors

In this study, family-related factors are caregiver education levels (high school or below vs. college or higher), only-child status (yes/no), parents' attitudes toward their children's consumption of SSBs (supportive, indifferent, non-supportive), and household availability of SSBs (yes/no).

#### 2.2.5 Body mass index

Height and weight were obtained from recent school physical examination records. Body mass index (BMI) was calculated as BMI = weight (kg)/ [height (m)]^2^. According to the criteria for screening for malnutrition and overweight and obesity among school-age children and adolescents in China (29, 30), students were classified into “ underweight “, “normal weight”, “overweight” and “obese”.

### 2.3 Quality control

A pilot survey in one primary, one junior high, and one senior high school ensured questionnaire clarity across age groups. For third-grade students, accuracy was enhanced through trained teacher assistance, beverage images as visual aids, a 7-day recall period, and immediate on-site checks for missing or inconsistent responses. All survey staff received standardized training on administration, ethics, and confidentiality. Data were double-entered independently in EpiData 3.1, with discrepancies resolved against original questionnaires.

### 2.4 Statistical analysis

All statistical analysis were performed using the statistical software IBM SPSS Statistics version 22.0. Continuous variables were expressed as mean and standard deviation. Categorical variables were expressed as count (*n*) and percentages (%). Comparisons between groups were made using a chi-square test. Multiple logistic regression analysis was used to examine the association between peer-related and family-related factors and SSBs intake, and demographic characteristics (sex, grade, place of residence, only-child status, caregiver's educational level) and health-related behaviors (daily water intake, outdoor activity time) were included as covariates. Statistical significance was defined as *p* < 0.05 (two-sided).

## 3 Results

### 3.1 Characteristics of participants

A total of 2,317 participants were included in the study, with a response rate of 91.8% (2,317/2,525), and all their data were available and included in the analysis. Table 1 shows the demographic characteristics of the participants and the consumption of SSBs over the past week. Among the 2,317 children, the mean age was 12.3 ± 2.9 years. Nearly half (48.7%) were boys, nearly half (48.2%) were from urban areas, 54.9% were only children, and 40.3 percent had a BMI of overweight (18.4 percent) or obese (21.9 %). The caregivers of the children were mainly parents (98.8%), grandparents (1.0%) or others (0.2%), and 81.9% of the caregivers had a college degree or above. In the past week, about 83.9% of children have consumed SSBs. Children who consumed SSBs were more likely to be older students, and caregivers were more likely to have a high school education or less than those who were non-consumer of SSBs (all *p* < 0.05).

**Table 1:** Demographic characteristics of participants and SSBs consumption in the past week: *n* (%).

**Characteristics**	**Total (*n =* 2,317)**	**SSBs** ^**b**^ **consumption**			
		**Yes (*****n** =* **1,945)**	**No (*****n** =* **375)**	χ^2^**/*****t***	***p*** **-value**
**Sex**				2.010	0.156
Boys	1,128 (48.7)	958 (84.9)	170 (15.1)		
Girls	1,189 (51.3)	984 (82.8)	205 (17.2)		
**Grade**				34.717	*p <* 0.001
Primary school (3th)	732 (31.6)	570 (77.9)	162 (22.1)		
Middle school (7th)	810 (35.0)	682 (84.2)	128 (15.8)		
High school (10th)	775 (33.4)	690 (89.0)	85 (11.0)		
**Region of residence**				0.132	0.717
Urban areas	1,117 (48.2)	933 (83.5)	184 (16.5)		
Suburb areas	1,200 (51.8)	1,009 (84.1)	191 (15.9)		
**Caregiver's educational level**				4.187	0.041
High school or below	420 (18.1)	366 (87.1)	54 (12.9)		
College or higher	1,897 (81.9)	1,576 (83.1)	321 (16.9)		
**Only-child status**				1.048	0.306
No	1,044 (45.1)	866 (83.0)	178 (17.0)		
Yes	1,273 (54.9)	1,076 (84.5)	197 (15.5)		
**BMI** ^a^				3.766	0.288
Underweight	138 (6.0)	116 (84.1)	22 (15.9)		
Normal	1,244 (53.7)	1,026 (82.5)	218 (17.5)		
Overweight	427 (18.4)	366 (85.7)	61 (14.3)		
Obese	508 (21.9)	434 (85.4)	74 (14.6)		

^a^BMI, body mass index; ^b^SSBs, 100% fruit/vegetable juices, fruit/vegetable beverages, carbonated beverages, tea beverages, milk beverages, plant protein drinks, beverages for special uses, coffee beverages.

### 3.2 SSBs consumption frequency

As shown in Table 2, about 81.9% of the participants had consumed pre-packaged sugary drinks in the past week. Fruit/vegetable beverages (36.3%), 100% fruit/vegetable juice (35.0%), and carbonated beverages (33.1%) were the most consumed beverages by primary school students, while coffee beverages (5.1%), plant protein beverages (20.9%), and beverages for special uses (24.2) were the least consumed. Tea beverages (48.8%), 100% fruit/vegetable juice (48.1%), and carbonated beverages (44.1%) were the most consumed beverages by middle school students, while coffee beverages (15.6%), plant protein beverages (22.0%), and milk beverages (34.0%) were the least consumed. For high school students, tea beverages (62.5%), carbonated beverages (56.0%), and 100% fruit/vegetable juice (46.4%) were the most consumed beverages, while plant protein beverages (28.3%), beverages for special uses (29.3%), and coffee beverages (34.6%) were the least consumed.

**Table 2:** Consumption of different types of prepackaged beverages by grade: *n* (%).

**Grade**	**Prepackaged beverages type**	**Consumption Frequency** ***n*** **(%)**		
		**Never consume**	**1–6 times/week**	≥**7 times/week**
Primary school (3th)	100% Fruit/vegetable juice	476 (65.0)	201 (27.5)	55 (7.5)
	Fruit/vegetable beverages^*^	466 (63.7)	221 (30.2)	45 (6.1)
	Carbonated beverages^*^	490 (66.9)	215 (29.4)	27 (3.7)
	Tea beverages^*^	496 (67.8)	199 (27.2)	37 (5.1)
	Milk beverages^*^	516 (70.5)	173 (23.6)	43 (5.9)
	Plant protein beverages^*^	579 (79.1)	107 (14.6)	46 (6.3)
	Beverages for special uses^*^	555 (75.8)	145 (19.8)	32 (4.4)
	Coffee beverages	695 (94.9)	30 (4.1)	7 (1.0)
Middle school (7th)	100% Fruit/vegetable juice	420 (51.9)	312 (38.5)	78 (9.6)
	Fruit/vegetable beverages	485 (59.9)	273 (33.7)	52 (6.4)
	Carbonated beverages	453 (55.9)	303 (37.4)	54 (6.7)
	Tea beverages	415 (51.2)	323 (39.9)	72 (8.9)
	Milk beverages	535 (66.0)	206 (25.4)	69 (8.5)
	Plant protein beverages	632 (78.0)	145 (17.9)	33 (4.1)
	Beverages for special uses	512 (63.2)	249 (30.7)	49 (6.0)
	Coffee beverages	684 (84.4)	99 (12.2)	27 (3.3)
High school (10th)	100% Fruit/vegetable juice	414 (53.4)	302 (39.0)	59 (7.6)
	Fruit/vegetable beverages	449 (57.9)	286 (36.9)	40 (5.2)
	Carbonated beverages	341 (44.0)	390 (50.3)	44 (5.7)
	Tea beverages	291 (37.5)	414 (53.4)	70 (9.0)
	Milk beverages	487 (62.8)	243 (31.4)	45 (5.8)
	Plant protein beverages	556 (71.7)	186 (24.0)	33 (4.3)
	Beverages for special uses	548 (70.7)	196 (25.3)	31 (4.0)
	Coffee beverages	507 (65.4)	217 (28.0)	51 (6.6)

^*^Fruit/vegetable beverages (vegetable- or fruit-flavored beverages that were not 100% fruit or vegetable juice. e.g., Minute Maid orange juice); Carbonated beverages (e.g., cola, Sprite); Tea beverages (e.g., iced tea, jasmine tea); Milk beverages (sugar-sweetened milk beverages that were not milk or yogurt. e.g., Fruity milk, Nutri-Express); Plant protein beverages (e.g., soya-bean milk drink, walnut drink, almond milk drink), Beverages for special uses (e.g., sports drinks, energy drinks, nutrient drinks, electrolyte drinks, such as Red Bull, Pulse).

### 3.3 Factors related to SSBs consumption

Table 3 displays that 76.0% of the participants had a daily water intake of 1,000 ml or more, and 52.8% had a daily outdoor activity time of 60 min or more. The majority of students (76.2%) did not get enough sleep during the week, and 73.0% of them had adequate sleep on the weekend. Sugar-sweetened beverage consumption was associated with higher daily water intake, peer-influenced purchase of SSBs, peer-sharing of SSBs, parental attitudes toward SSBs and household availability (all *p* < 0.05). With the exception of carbonated beverages, plant protein beverages and beverages for special uses, the other five types of SSBs were associated with higher daily water intake, peer-influenced purchase of SSBs, peer-sharing of SSBs, parental attitudes toward SSBs and household availability (all *p* < 0.05).

**Table 3:** Relationships between health-related behaviors, peer-related factors, home-related factors and SSBs consumption.

**Relevant factors**	**SSBs consumption**			
	**Yes (*****n** =* **1,945)**	**No (*****n** =* **375)**	χ^2^**/*****t***	***p*** **-value**
Daily water intake			9.217	0.010
<1,000 ml/day	444 (80.0)	111 (20.0)		
1,000–1,500 ml/day	649 (83.9)	125 (16.1)		
>1,500 ml/day	849 (85.9)	139 (14.1)		
Outdoor activity time			0.065	0.798
<60 min/day	913 (83.6)	179 (16.4)		
≥60 min/day	1,029 (84.0)	196 (16.0)		
Weekday sleep time			2.384	0.123
Insufficient	1,491 (84.5)	274 (15.5)		
Sufficient	451 (81.7)	101 (18.3)		
Weekend sleep time			1.312	0.252
Insufficient	514 (82.4)	110 (17.6)		
Sufficient	1,428 (84.3)	265 (15.7)		
Peer-influenced purchase of SSBs			45.609	*p <* 0.001
No	715 (77.5)	208 (22.5)		
Yes	1,227 (88.0)	167 (12.0)		
Peer-sharing of SSBs			78.327	*p <* 0.001
No	509 (73.4)	184 (26.6)		
Yes	1,433 (88.2)	191 (11.8)		
Parents' attitudes toward SSBs			38.563	*p <* 0.001
Supportive	225 (86.5)	35 (13.5)		
Non-supportive	944 (79.3)	247 (20.7)		
Indifferent	733 (86.3)	93 (10.7)		
Household availability of SSBs			35.143	*p <* 0.001
No	656 (77.8)	187 (22.2)		
Yes	1,286 (87.2)	188 (12.8)		

### 3.4 Associations between SSBs consumption and relevant factors

As shown in Table 4, after adjusting for sex, grade, residence, only-child status, caregiver education, daily water intake, and outdoor activity time, logistic regression showed that peer-influenced purchasing, peer sharing, supportive or indifferent parental attitudes, and household SSB availability were all significantly associated with higher odds of consuming most SSB categories. Taking fruit/vegetable beverages as an example, students who purchase sugar drinks under peer-influenced (OR: 1.600,95% CI:1.318–1.941) and share SSBs with their peers (OR: 1.373,95% CI:1.106–1.704) were more likely to consume fruit/vegetable beverages than the counterparts. When there was a household availability of SSBs environment (OR: 1.305, 95% CI:1.085–1.570), children were more likely consume fruit/vegetable beverages compared with the “No” group. In addition, the children whose parents' attitudes toward SSBs “supportive” (OR: 2.246, 95% CI:1.691–2.981), or “indifferent” (OR: 1.573,95% CI: 1.295–1.910) were more likely to consume fruit/vegetable beverages compared with the “non-supportive” group. Similar associations were observed for carbonated beverages, tea beverages, milk beverages, and other categories, though effect sizes varied.

**Table 4:** Associations between consumption of different types of SSBs and influencing factors: multivariate analysis.

**Variable**	**100% Fruit/vegetable juice**		**Fruit/vegetable beverages** ^*^	
	**OR (95% CI)**	***p*** **-value**	**OR (95% CI)**	***p*** **-value**
**Boys** (reference: girls)	0.871 (0.733–1.036)	0.118	1.359 (1.136–1.624)	0.001
**Grade**				
Middle school (reference: primary school)	1.501 (1.208–1.866)	*p <* 0.001	1.022 (0.816–1.280)	0.852
High school (reference: primary school)	1.440 (1.147–1.807)	0.002	0.937 (0.741–1.184)	0.586
**Suburb areas** (reference: urban areas)	1.043 (0.879–1.238)	0.629	1.318 (1.104–1.573)	0.002
**Only-child status** (reference: no)	1.073 (0.903–1.274)	0.426	0904 (0.756–1.080)	0.266
**Caregiver's educational level**				
High school or below (reference: College or higher)	0.917 (0.734–1.147)	0.449	1.145 (0.911–1.438)	0.247
**Daily water intake**				
1,000–1,500 ml/day (reference: < 1,000 ml/day)	1.194 (0.948–1.504)	0.131	1.438 (1.133–1.824)	0.003
>1,500 ml/day (reference: < 1,000 ml/day)	1.465 (1.166–1.841)	0.001	1.344 (1.059–1.705)	0.015
Outdoor activity time ≥60 min/day (reference: < 60 min/day)	1.382 (1.159–1.649)	*p <* 0.001	0.938 (0.782–1.125)	0.492
**Peer-influenced purchase of SSBs** (reference: no)	1.189 (0.986–1.433)	0.070	1.600 (1.318–1.941)	*p <* 0.001
**Peer-sharing of SSBs** (reference: no)	1.377 (1.120–1.693)	0.002	1.373 (1.106–1.704)	0.004
**Parents' attitudes toward SSBs**				
Supportive (reference: non-supportive)	1.354 (1.024–1.790)	0.033	2.246 (1.691–2.981)	*p <* 0.001
Indifferent (reference: non-supportive)	0.916 (0.758–1.107)	0.364	1.573 (1.295–1.910)	*p <* 0.001
**Household availability of SSBs** (reference: no)	1.305 (1.085–1.570)	0.005	1.644 (1.356–1.993)	*p <* 0.001
**Variable**	**Carbonated beverages** ^*^		**Tea beverages** ^*^	
	**OR (95% CI)**	***p*** **-value**	**OR (95% CI)**	***p*** **-value**
**Boys** (reference: girls)	2.227 (1.852–2.678)	*p <* 0.001	1.224 (1.023–1.465)	0.027
**Grade**				
Middle school (reference: primary school)	1.538 (1.221–1.937)	*p <* 0.001	1.755 (1.402–2.197)	*p <* 0.001
High school (reference: primary school)	2.103 (1.658–2.669)	*p <* 0.001	2.644 (2.092–3.341)	*p <* 0.001
**Suburb areas** (reference: urban areas)	1.308 (1.093–1.566)	0.003	1.455 (1.219–1.738)	*p <* 0.001
**Only-child status** (reference: no)	0.784 (0.654–0.940)	0.009	0.912 (0.763–1.091)	0.314
**Caregiver's educational level**				
High school or below (reference: College or higher)	1.124 (0.890–1.418)	0.0326	1.368 (1.085–1.725)	0.008
**Daily water intake**				
1,000–1,500 ml/day (reference: < 1,000 ml/day)	1.020 (0.802–1.296)	0.874	1.441 (1.136–1.829)	0.003
>1,500 ml/day (reference: < 1,000 ml/day)	0.915 (0.720–1.163)	0.467	1.311 (1.035–1.662)	0.025
Outdoor activity time ≥60 min/day (reference: < 60 min/day)	0.896 (0.744–1.078)	0.243	1.010 (0.841–1.213)	0.913
**Peer-influenced purchase of SSBs** (reference: no)	1.768 (1.454–2.149)	*p <* 0.001	1.424 (1.175–1.725)	*p <* 0.001
**Peer-sharing of SSBs** (reference: no)	1.514 (1.218–1.882)	*p <* 0.001	1.662 (1.345–2.056)	*p <* 0.001
**Parents' attitudes toward SSBs**				
Supportive (reference: non-supportive)	1.634 (1.224–2.181)	0.001	1.484 (1.112–1.981)	0.007
Indifferent (reference: non-supportive)	1.944 (1.597–2.367)	*p <* 0.001	1.590 (1.310–1.929)	*p <* 0.001
Household availability of SSBs (reference: no)	1.515 (1.249–1.839)	*p <* 0.001	1.408 (1.164–1.703)	*p <* 0.001
**Variable**	**Milk beverages** ^*^		**Plant protein beverages** ^*^	
	**OR (95% CI)**	***p*** **-value**	**OR (95% CI)**	***p*** **-value**
**Boys** (reference: girls)	0.934 (0.781–1.118)	0.459	1.140 (0.934–1.391)	0.196
**Grade**				
Middle school (reference: primary school)	1.095 (0.872–1.376)	0.434	0.929 (0.718–1.202)	0.574
High school (reference: primary school)	1.222 (0.964–1.547)	0.097	1.339 (1.033–1.737)	0.028
**Suburb areas** (reference: urban areas)	1.037 (0.868–1.239)	0.690	0.895 (0.735–1.091)	0.272
**Only-child status** (reference: no)	0.931 (0.778–1.114)	0.434	0.956 (0.784–1.166)	0.658
**Caregiver's educational level**				
High school or below (reference: College or higher)	1.163 (0.926–1.461)	0.194	1.126 (0.887–1.446)	0.352
**Daily water intake**				
1,000–1,500 ml/day (reference: < 1,000 ml/day)	0.940 (0.739–1.195)	0.614	1.119 (0.852–1.470)	0.418
>1,500 ml/day (reference: < 1,000 ml/day)	1.139 (0.900–1.443)	0.279	1.285 (0.984–1.679)	0.065
Outdoor activity time ≥60 min/day (reference: < 60 min/day)	1.242 (1.033–1.493)	0.021	1.409 (1.148–1.730)	0.001
**Peer-influenced purchase of SSBs** (reference: no)	1.252 (1.030–1.523)	0.024	1.044 (0.841–1.297)	0.694
**Peer-sharing of SSBs** (reference: no)	1.400 (1.124–1.744)	0.003	1.523 (1.189–1.950)	0.001
**Parents' attitudes toward SSBs**				
Supportive (reference: non-supportive)	1.418 (1.069–1.881)	0.015	1.119 (0.817–1.533)	0.484
Indifferent (reference: non-supportive)	0.943 (0.773–1.150)	0.563	0.896 (0.719–1.117)	0.330
**Household availability of SSBs** (reference: no)	1.264 (1.041–1.534)	0.018	0.969 (0.784–1.198)	0.774
**Variable**	**Beverages for special uses** ^*^		**Coffee beverages**	
	**OR (95% CI)**	***p*** **-value**	**OR (95% CI)**	***p*** **-value**
**Boys** (reference: girls)	1.875 (1.554–2.262)	*p <* 0.001	1.103 (0.875–1.390)	0.405
**Grade**				
Middle school (reference: primary school)	1.629 (1.286–2.065)	*p <* 0.001	3.097 (2.092–4.858)	*p <* 0.001
High school (reference: primary school)	1.158 (0.899–1.492)	0.225	8.223 (5.635–12.001)	*p <* 0.001
**Suburb areas** (reference: urban areas)	1.174 (0.975–1.414)	0.091	1.065 (0.849–1.336)	0.584
**Only-child status** (reference: no)	0.961 (0.797–1.159)	0.676	0.966 (0.768–1.214)	0.765
**Caregiver's educational level**				
High school or below (reference: college or higher)	1.049 (0.825–1.332)	0.698	1.054 (0.803–1.382)	0.706
**Daily water intake**				
1,000–1,500 ml/day (reference: < 1,000 ml/day)	0.953 (0.737–1.233)	0.716	0.980 (0.715–1.343)	0.900
>1,500 ml/day (reference: < 1,000 ml/day)	1.225 (0.955–1.572)	0.111	1.088 (0.800–1.479)	0.590
Outdoor activity time ≥60 min/day (reference: < 60 min/day)	1.550 (1.279–1.879)	*p <* 0.001	0.983 (0.777–1.244)	0.887
**Peer-influenced purchase of SSBs** (reference: no)	1.210 (0.987–1.483)	0.067	1.111 (0.862–1.431)	0.416
**Peer-sharing of SSBs** (reference: no)	1.181 (0.943–1.479)	0.148	1.329 (0.984–1.793)	0.064
**Parents' attitudes toward SSBs**				
Supportive (reference: non-supportive)	0.907 (0.664–1.239)	0.540	1.719 (1.211–2.440)	0.002
Indifferent (reference: non-supportive)	1.175 (0.958–1.442)	0.110	1.516 (1.184–1.942)	0.001
**Household availability of SSBs** (reference: no)	1.162 (0.950–1.421)	0.145	1.283 (0.998–1.650)	0.052

Adjusted for confounding factors such as gender, grade, region of residence, whether the child was the only one in the family, caregiver's education level, daily water intake and outdoor activity time. ^*^Fruit/vegetable beverages (vegetable- or fruit-flavored beverages that were not 100% fruit or vegetable juice. e.g., Minute Maid orange juice); Carbonated beverages (e.g., cola, Sprite); Tea beverages (e.g., iced tea, jasmine tea); Milk beverages (sugar-sweetened milk beverages that were not milk or yogurt. e.g., Fruity milk, Nutri-Express); Plant protein beverages (e.g., soya-bean milk drink, walnut drink, almond milk drink), Beverages for special uses (e.g., sports drinks, energy drinks, nutrient drinks, electrolyte drinks, such as Red Bull, Pulse).

## 4 Discussion

This large, school-based cross-sectional study found that more than four-fifths (81.9%) of primary and secondary school students in Beijing consumed at least one type of pre-packaged SSB in the past week.

One previous nation study found that the intake of SSBs in Chinese children aged 6–17 years in 2012 was estimated at 181.0 g/day, occurring 2.2 times per week (31). Data from the 2014 National Intervention Program for Obesity in Children and Adolescents Aged 6–17 Years in China showed that 66.6% of participants reported consuming sugar-sweetened beverages (32). This result echoes previous research in Beijing, China. For example, a cross-sectional study of preschoolers in Dongcheng District, Beijing, China, found that about 84.5% of pre-schoolers had consumed SSB in the past 3 months (33), which included milk beverages, fruit/vegetable beverages, vegetable protein beverages, carbonated beverages, tea beverages, and sports/energy beverages, a total of 6 beverages. Our study added 100% fruit/vegetable juices and coffee beverages to a total of 8 types. This study found that the proportion of primary and secondary school students in Beijing who consumed SSBs was higher than in other parts of China. This difference may reflect Beijing's uniqueness as a metropolis in terms of food culture and lifestyle.

The study also found that students consume SSB more frequently with age, which is consistent with some international studies. According to the National Health and Nutrition Examination Survey (NHANES), older adolescents have the highest average intake and percentage of daily calories from SSBs than younger children (34). In addition, the prevalence of high-added sugar consumers is significantly higher among those aged 12–19 years compared to those aged 2–5 years (35). According to a study in the United States (36), the percent energy contributed by added sugars was 14.3 ± 0.2% (2–8 years), 16.2 ± 0.2% (9–18 years), and 13.1 ± 0.2% (≥19 years), suggesting the highest intakes are among adolescents and teens. Tea drinks are more common among secondary school students, while primary school students are more likely to choose fruit and vegetable beverages. This may be due to the misconception that fruit and vegetable juice drinks are healthier in younger children. However, in the survey of the sugar content of beverages in the Beijing market, fruit and vegetable juice drinks had the highest sugar content (10.0 g/100 ml) (37). Older students have greater autonomy in their dietary choices and are more influenced by advertising and social media (38–40), leading to differences in sugary beverage preferences. In addition, different stages of mental and physical development may also have an impact on beverage preference and motivation to drink, which can be explained by the theory of self-determination (41, 42). Carbonated beverages are popular among young people all over the world (43, 44). Hundred percentage fruit and vegetable juices have also been shown to be associated with increased BMI in children (45). However, a study found that the consumption of herbal teas and dietary beverages increased and the eating habits of students generally changed positively (13), suggesting that we can replace high-sugar drinks with sugar-free or low-sugar drinks to reduce added sugar intake.

This study further found that peer purchasing and sharing behaviors significantly increased the likelihood of SSBs intake among students. This result is consistent with the social norm theory, which states that adolescents tend to imitate group behavior to gain a sense of belonging and identity (46). In school, peer interaction is frequent, and drink sharing may be seen as part of social interaction, thus invisibly driving the popularity of SSBs. Previous studies have found that the establishment of positive “peer influence mechanisms”, such as student health advocacy groups and social network interventions, should be considered when designing interventions (47, 48).

This study found that the availability of SSBs in the household was significantly associated with SSBs intake in children, which was consistent with previous studies in Beijing. They pointed out that children's eating behavior is heavily influenced by family food availability, eating habits, eating rules, and parental nutritional literacy (49). In addition, when parents clearly expressed their disapproval of SSBs consumption, students' SSBs intake levels were significantly reduced. This also validates the important role of the family in the formation of children's health behaviors (22, 50), emphasizing the key role of parents as “gatekeepers of health behaviors”.

The results of this study suggest that concerted efforts should be made at the school, family and social levels to reduce the intake of SSBs among primary and secondary school students (51). It is suggested that schools should strengthen healthy diet education and limit the sale and promotion of sugary drinks on campus (52, 53). Families should reduce the stockpile of SSBs at home, and parents should set an example of healthy eating. A combination of school-based and home-based interventions appears to be effective in reducing sugar-sweetened beverage consumption among schoolchildren in China (54). Society can legislate to restrict the advertising of SSBs targeting minors (55, 56). Peer role plays an important role in adolescents' food choices, so it is important to encourage the formation of a peer culture of “healthy eating” (57). Future studies can use a longitudinal tracking design to evaluate the effects of long-term intake of SSBs on weight gain, metabolic indexes, and psychological and behavioral development. In addition, the impact of broader socio-ecological factors such as media exposure, school nutrition policy, and community environment on SSBs consumption should be further explored to guide the formulation of intervention strategies more comprehensively.

Although this study provides new evidence on the influencing factors of SSBs consumption among school-age children in China, the following limitations should be noted. First, this study used a self-report questionnaire to collect data, which may lead to underestimation or overestimation of the frequency of consumption of sugar-sweetened beverages and exposure to related influencing factors. Second, this study is a cross-sectional study, and the causal relationship of potential confounders cannot be inferred. Third, Beijing is a representative of China's advanced economic cities, which could lead to bias in comparison with the rest of China.

## 5 Conclusions

This study shows that the consumption rate of SSBs among primary and secondary school students is relatively high in Beijing. Our findings highlight the role of peer and family-related factors in SSBs consumption. Exposure to peer-related factors, such as peer-influenced purchasing behavior, peer-sharing behavior, and availability of SSBs in the home setting, were associated with a higher likelihood of consumption of SSBs in children. In contrast, parental disapproval or neutral attitudes toward SSBs were negatively correlated with SSBs consumption. Therefore, interventions should combine school-level actions (ban SSB sales in canteens, strengthen peer-led health promotion) with family-level strategies (parental education, limiting home availability) and policy measures (warning labels on high-sugar beverages, restricting advertising to minors).to reduce SSBs consumption.

## Data Availability

The original contributions presented in the study are included in the article/Supplementary material, further inquiries can be directed to the corresponding author.
