# Correction: Association between peer behaviors and family environment and pre-packaged sugar-sweetened beverage consumption among primary and secondary school students in Beijing

**DOI:** 10.3389/fpubh.2025.1718297

**Published:** 2025-10-16

**Authors:** Yiran Li, Lulu Meng, Siyu Liang, Yan Zhang, Wenjia Li, Jiali Duan, Ruoxiang Cao, Jie Li, Liyu Huang

**Affiliations:** ^1^School of Public Health, Hebei Medical University, Shijiazhuang, Hebei, China; ^2^Office of Research and Teaching Administration, Beijing Center for Disease Prevention and Control, Beijing, China; ^3^Institute of Nutrition and Food Hygiene, Fengtai District Center for Disease Prevention and Control, Beijing, China; ^4^Institute of Nutrition and Food Hygiene, Beijing Center for Disease Prevention and Control, Beijing, China; ^5^School of Public Health, Capital Medical University, Beijing, China

**Keywords:** sugar-sweetened beverages, children, peer, family, school-age

Affiliation “Institute of Nutrition and Food Hygiene, Beijing Center for Disease Prevention and Control, Beijing, China” was omitted for author Liyu Huang. This affiliation has now been added for author Liyu Huang.

The original version of this article has been updated.

