# Supplementary material for: Association between peer behaviors and family environment and pre-packaged sugar-sweetened beverage consumption among primary and secondary school students in Beijing
Source: Front Public Health. 2025 Oct 1;13:1661141. doi: 10.3389/fpubh.2025.1661141 (PMC12521133; doi:10.3389/fpubh.2025.1661141)
Supplement: Supplementary file 1 [file Table_1.docx]

Supplementary Material

# Supplementary Figure

**
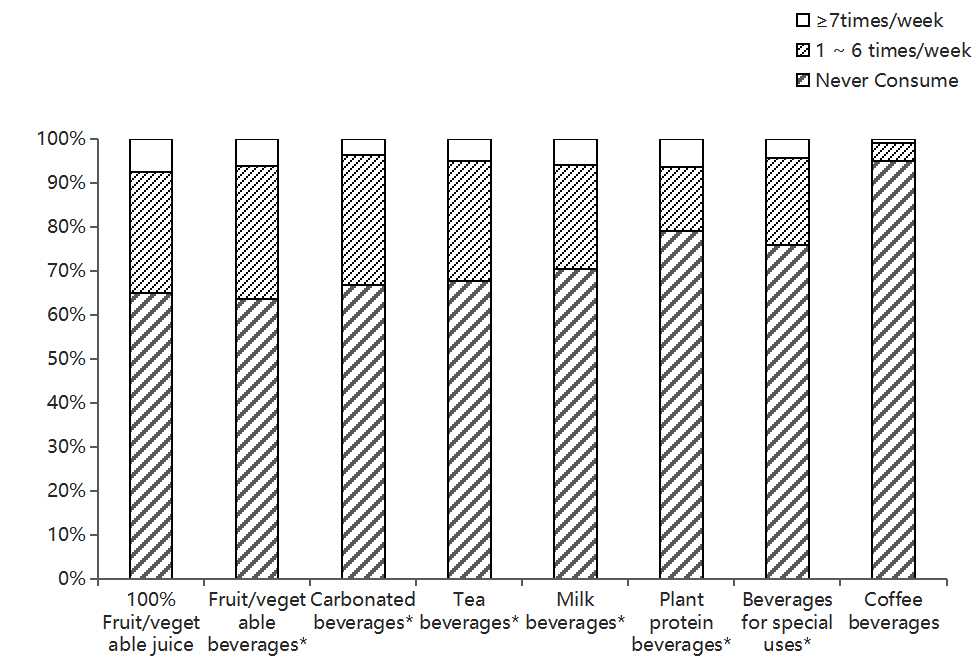
**

**Supplementary Figure 1.** Consumption of different types of prepackaged beverages by primary school students

Notes: *: Fruit/vegetable beverages (vegetable- or fruit-flavored beverages that were not 100% fruit or vegetable juice. e.g., Minute Maid orange juice); Carbonated beverages (e.g., cola, Sprite); Tea beverages (e.g., iced tea, jasmine tea); Milk beverages (sugar-sweetened milk beverages that were not milk or yogurt. e.g., Fruity milk, Nutri-Express); Plant protein beverages (e.g., soya-bean milk drink, walnut drink, almond milk drink), Beverages for special uses (e.g., sports drinks, energy drinks, nutrient drinks, electrolyte drinks, such as Red Bull, Pulse).

**
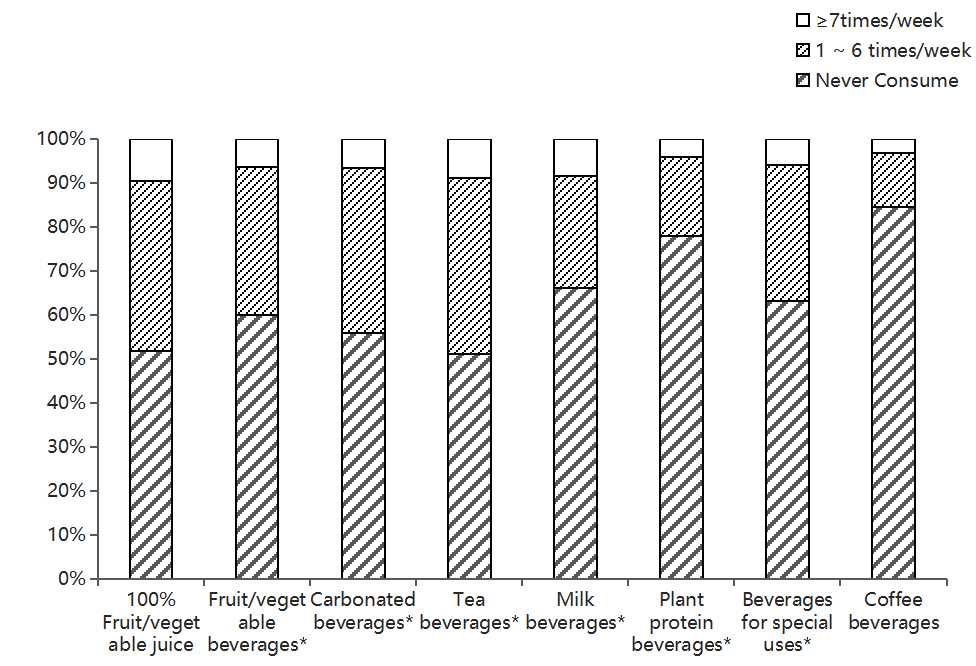
**

**Supplementary Figure 2.** Consumption of different types of prepackaged beverages by middle school students

Notes: *: Fruit/vegetable beverages (vegetable- or fruit-flavored beverages that were not 100% fruit or vegetable juice. e.g., Minute Maid orange juice); Carbonated beverages (e.g., cola, Sprite); Tea beverages (e.g., iced tea, jasmine tea); Milk beverages (sugar-sweetened milk beverages that were not milk or yogurt. e.g., Fruity milk, Nutri-Express); Plant protein beverages (e.g., soya-bean milk drink, walnut drink, almond milk drink), Beverages for special uses (e.g., sports drinks, energy drinks, nutrient drinks, electrolyte drinks, such as Red Bull, Pulse).

**
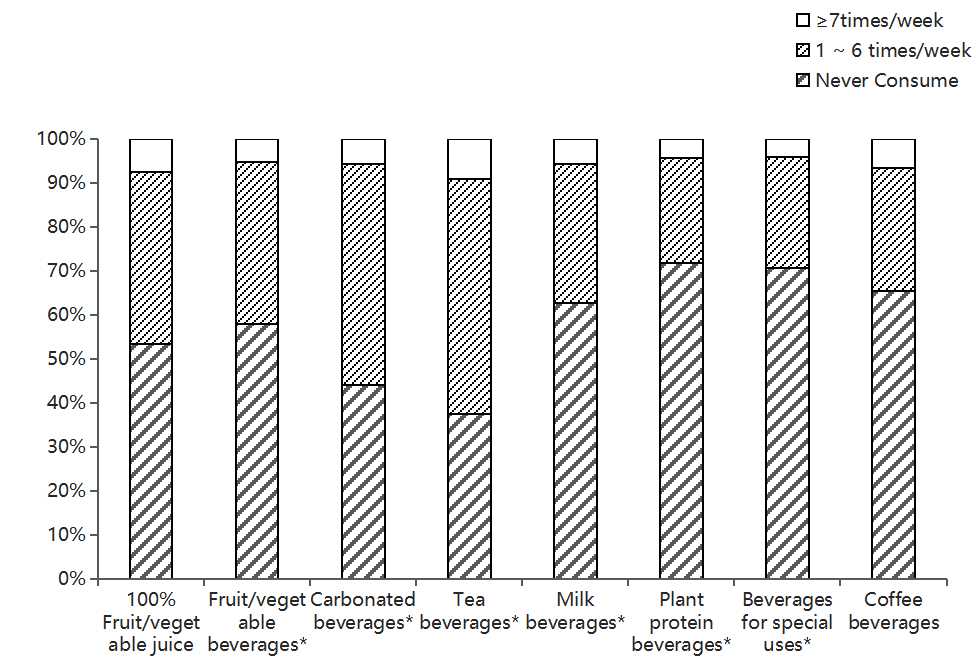
**

**Supplementary Figure 3.** Consumption of different types of prepackaged beverages by high school students

Notes: *: Fruit/vegetable beverages (vegetable- or fruit-flavored beverages that were not 100% fruit or vegetable juice. e.g., Minute Maid orange juice); Carbonated beverages (e.g., cola, Sprite); Tea beverages (e.g., iced tea, jasmine tea); Milk beverages (sugar-sweetened milk beverages that were not milk or yogurt. e.g., Fruity milk, Nutri-Express); Plant protein beverages (e.g., soya-bean milk drink, walnut drink, almond milk drink), Beverages for special uses (e.g., sports drinks, energy drinks, nutrient drinks, electrolyte drinks, such as Red Bull, Pulse).

# Supplementary Tables

**Supplementary Table 1.** Relationships between health-related behaviors, peer-related factors, home-related factors and 100% fruit/vegetable juice consumption

| **Variables** | **100% Fruit/vegetable juice Consumption(n=2317)** | | | |
| --- | --- | --- | --- | --- |
|  | **Yes**  **(n=1007)** | **No**  **(n=1310)** | **χ^2^/t** | ***p*-Value** |
| **Daily water intake** |  |  | 25.420 | *p*＜0.001 |
| ＜1000 ml/day | 202(36.4) | 353(63.6) |  |  |
| 1000~1500 ml/day | 320(41.3) | 454(58.7) |  |  |
| ＞1500 ml/day | 485(49.1) | 503(50.9) |  |  |
| **Outdoor activity time** |  |  | 17.340 | *p*＜0.001 |
| ＜60 minutes/day | 425(38.9) | 667(61.1) |  |  |
| ≥60 minutes/day | 582(47.5) | 643(52.5 |  |  |
| **Weekday sleep time** |  |  | 8.192 | 0.004 |
| Insufficient | 738(41.8) | 1027(58.2) |  |  |
| Sufficient | 269(48.7) | 283(51.3) |  |  |
| **Weekend sleep time** |  |  | 10.439 | 0.001 |
| Insufficient | 237(38.0) | 387(62.0) |  |  |
| Sufficient | 770(45.5) | 923(54.5) |  |  |
| **Peer-influenced purchase of SSBs** |  |  | 11.813 | 0.001 |
| No | 361(39.1) | 562(60.9) |  |  |
| Yes | 646(46.3) | 748(53.7) |  |  |
| **Peer-sharing of SSBs** |  |  | 29.350 | *p*＜0.001 |
| No | 242(34.9) | 451(65.1) |  |  |
| Yes | 765(47.1) | 859(52.9) |  |  |
| **Parents’ attitudes toward SSBs** |  |  | 8.357 | 0.015 |
| Supportive | 134(51.5) | 126(48.5) |  |  |
| Non-supportive | 497(41.7) | 694(58.3) |  |  |
| Indifferent | 376(43.4) | 490(56.6) |  |  |
| **Household availability of SSBs** |  |  | 15.627 | *p*＜0.001 |
| No | 321(38.1) | 522(61.9) |  |  |
| Yes | 686(46.5) | 788(53.5) |  |  |

**Supplementary Table 2.** Relationships between health-related behaviors, peer-related factors, home-related factors and fruit/vegetable beverages consumption

| **Variables** | **Fruit/vegetable beverages Consumption(n=2317)** | | | |
| --- | --- | --- | --- | --- |
|  | **Yes**  **(n=917)** | **No**  **(n=1400)** | **χ^2^/t** | ***p*-Value** |
| **Daily water intake** |  |  | 12.364 | 0.002 |
| ＜1000 ml/day | 186(33.5) | 369(66.5) |  |  |
| 1000~1500 ml/day | 332(42.9) | 442(57.1) |  |  |
| ＞1500 ml/day | 399(40.4) | 589(42.1) |  |  |
| **Outdoor activity time** |  |  | 1.383 | 0.240 |
| ＜60 minutes/day | 446(40.8) | 646(59.2) |  |  |
| ≥60 minutes/day | 471(38.4) | 754(61.6) |  |  |
| **Weekday sleep time** |  |  | 0.416 | 0.519 |
| Insufficient | 705(39.9) | 1060(60.1) |  |  |
| Sufficient | 212(38.4) | 340(61.6) |  |  |
| **Weekend sleep time** |  |  | 4.060 | 0.044 |
| Insufficient | 268(42.9) | 356(57.1) |  |  |
| Sufficient | 649(38.3) | 1044(61.7) |  |  |
| **Peer-influenced purchase of SSBs** |  |  | 57.386 | *p*＜0.001 |
| No | 278(30.1) | 645(69.9) |  |  |
| Yes | 639(45.8) | 755(54.2) |  |  |
| **Peer-sharing of SSBs** |  |  | 41.308 | *p*＜0.001 |
| No | 205(29.6) | 488(70.4) |  |  |
| Yes | 712(43.8) | 912(56.2) |  |  |
| **Parents’ attitudes toward SSBs** |  |  | 65.046 | *p*＜0.001 |
| Supportive | 137(52.7) | 123(47.3) |  |  |
| Non-supportive | 379(31.8) | 812(68.2) |  |  |
| Indifferent | 401(46.3) | 465(53.7) |  |  |
| **Household availability of SSBs** |  |  | 59.883 | *p*＜0.001 |
| No | 246(29.2) | 597(70.8) |  |  |
| Yes | 671(45.5) | 803(54.5) |  |  |

**Supplementary Table 3.** Relationships between health-related behaviors, peer-related factors, home-related factors and carbonated beverages consumption

| **Variables** | **Carbonated beverages Consumption(n=2317)** | | | |
| --- | --- | --- | --- | --- |
|  | **Yes**  **(n=1033)** | **No**  **(n=1284)** | **χ^2^/t** | ***p*-Value** |
| **Daily water intake** |  |  | 2.446 | 0.294 |
| ＜1000 ml/day | 232(41.8) | 323(58.2) |  |  |
| 1000~1500 ml/day | 356(46.0) | 418(54.0) |  |  |
| ＞1500 ml/day | 445(45.0) | 543(55.0) |  |  |
| **Outdoor activity time** |  |  | 4.434 | 0.035 |
| ＜60 minutes/day | 512(46.9) | 580(53.1) |  |  |
| ≥60 minutes/day | 521(42.5) | 704(57.5) |  |  |
| **Weekday sleep time** |  |  | 0.982 | 0.322 |
| Insufficient | 797(45.2) | 968(54.8) |  |  |
| Sufficient | 236(42.8) | 316(57.2 |  |  |
| **Weekend sleep time** |  |  | 0 | 0.985 |
| Insufficient | 278(44.6) | 346(55.4) |  |  |
| Sufficient | 755(44.6) | 938(55.4) |  |  |
| **Peer-influenced purchase of SSBs** |  |  | 87.402 | *p*＜0.001 |
| No | 302(32.7) | 621(67.3) |  |  |
| Yes | 731(52.4) | 663(47.6) |  |  |
| **Peer-sharing of SSBs** |  |  | 67.442 | *p*＜0.001 |
| No | 219(31.6) | 474(68.4) |  |  |
| Yes | 814(50.1) | 810(49.9) |  |  |
| **Parents’ attitudes toward SSBs** |  |  | 95.128 | *p*＜0.001 |
| Supportive | 131(50.4) | 129(49.6) |  |  |
| Non-supportive | 416(34.9) | 775(65.1) |  |  |
| Indifferent | 486(56.1) | 380(43.9) |  |  |
| **Household availability of SSBs** |  |  | 55.611 | *p*＜0.001 |
| No | 290(34.4) | 553(65.6) |  |  |
| Yes | 743(50.4) | 731(49.6) |  |  |

**Supplementary Table 4.** Relationships between health-related behaviors, peer-related factors, home-related factors and tea beverages consumption

| **Variables** | **Tea beverages Consumption(n=2317)** | | | |
| --- | --- | --- | --- | --- |
|  | **Yes**  **(n=1115)** | **No**  **(n=1202)** | **χ^2^/t** | ***p*-Value** |
| **Daily water intake** |  |  | 21.958 | *p*＜0.001 |
| ＜1000 ml/day | 219(39.5) | 336(60.5) |  |  |
| 1000~1500 ml/day | 395(51.0) | 379(49.0) |  |  |
| ＞1500 ml/day | 501(50.7) | 487(49.3) |  |  |
| **Outdoor activity time** |  |  | 0.918 | 0.338 |
| ＜60 minutes/day | 537(49.2) | 555(50.8) |  |  |
| ≥60 minutes/day | 578(47.2) | 647(52.8) |  |  |
| **Weekday sleep time** |  |  | 0.018 | 0.894 |
| Insufficient | 848(48.0) | 917(52.0) |  |  |
| Sufficient | 267(48.4) | 285(51.6) |  |  |
| **Weekend sleep time** |  |  | 4.363 | 0.037 |
| Insufficient | 278(44.6) | 346(55.4) |  |  |
| Sufficient | 837(49.4) | 856(50.6) |  |  |
| **Peer-influenced purchase of SSBs** |  |  | 54.813 | *p*＜0.001 |
| No | 357(38.7) | 566(61.3) |  |  |
| Yes | 758(54.4) | 636(45.6) |  |  |
| **Peer-sharing of SSBs** |  |  | 88.323 | *p*＜0.001 |
| No | 230(33.2) | 463(66.8) |  |  |
| Yes | 885(54.5) | 739(45.5) |  |  |
| **Parents’ attitudes toward SSBs** |  |  | 78.272 | *p*＜0.001 |
| Supportive | 140(53.8) | 120(46.2) |  |  |
| Non-supportive | 468(39.3) | 723(60.7) |  |  |
| Indifferent | 207(58.5) | 359(41.5) |  |  |
| **Household availability of SSBs** |  |  | 39.448 | *p*＜0.001 |
| No | 333(39.5) | 510(60.5) |  |  |
| Yes | 782(53.1) | 692(46.9) |  |  |

**Supplementary Table 5.** Relationships between health-related behaviors, peer-related factors, home-related factors and milk beverages consumption

| **Variables** | **Milk beverages Consumption(n=2317)** | | | |
| --- | --- | --- | --- | --- |
|  | **Yes**  **(n=779)** | **No**  **(n=1538)** | **χ^2^/t** | ***p*-Value** |
| **Daily water intake** |  |  | 6.248 | 0.044 |
| ＜1000 ml/day | 178(32.1) | 377(67.9) |  |  |
| 1000~1500 ml/day | 241(31.1) | 533(68.9) |  |  |
| ＞1500 ml/day | 360(36.4) | 628(63.6) |  |  |
| **Outdoor activity time** |  |  | 5.304 | 0.021 |
| ＜60 minutes/day | 341(31.2) | 751(68.8) |  |  |
| ≥60 minutes/day | 438(35.8) | 787(64.2) |  |  |
| **Weekday sleep time** |  |  | 1.642 | 0.200 |
| Insufficient | 581(32.9) | 1184(67.1) |  |  |
| Sufficient | 198(35.9) | 354(64.1) |  |  |
| **Weekend sleep time** |  |  | 6.539 | 0.011 |
| Insufficient | 184(29.5) | 440(70.5) |  |  |
| Sufficient | 595(35.1) | 1098(64.9) |  |  |
| **Peer-influenced purchase of SSBs** |  |  | 16.575 | *p*＜0.001 |
| No | 265(28.7) | 658(71.3) |  |  |
| Yes | 514(36.9) | 880(63.1) |  |  |
| **Peer-sharing of SSBs** |  |  | 25.907 | *p*＜0.001 |
| No | 180(26.0) | 513(74.0) |  |  |
| Yes | 599(36.9) | 1025(63.1) |  |  |
| **Parents’ attitudes toward SSBs** |  |  | 10.385 | 0.006 |
| Supportive | 110(42.3) | 150(57.7) |  |  |
| Non-supportive | 380(31.9) | 811(68.1) |  |  |
| Indifferent | 289(33.4) | 577(66.6) |  |  |
| **Household availability of SSBs** |  |  | 12.987 | *p*＜0.001 |
| No | 244(28.9) | 599(71.1) |  |  |
| Yes | 535(36.3) | 939(63.7) |  |  |

**Supplementary Table 6.** Relationships between health-related behaviors, peer-related factors, home-related factors and plant protein beverages consumption

| **Variables** | **Plant protein beverages Consumption(n=2317)** | | | |
| --- | --- | --- | --- | --- |
|  | **Yes**  **(n=550)** | **No**  **(n=1767)** | **χ^2^/t** | ***p*-Value** |
| **Daily water intake** |  |  | 9.893 | 0.007 |
| ＜1000 ml/day | 112(20.2) | 443(79.8) |  |  |
| 1000~1500 ml/day | 173(22.4) | 601(77.6) |  |  |
| ＞1500 ml/day | 265(26.8) | 723(73.2) |  |  |
| **Outdoor activity time** |  |  | 13.973 | *p*＜0.001 |
| ＜60 minutes/day | 221(20.2) | 871(79.8) |  |  |
| ≥60 minutes/day | 329(26.9) | 896(73.1) |  |  |
| **Weekday sleep time** |  |  | 6.340 | 0.012 |
| Insufficient | 397(22.5) | 1368(77.5) |  |  |
| Sufficient | 153(27.7) | 399(72.3) |  |  |
| **Weekend sleep time** |  |  | 8.912 | 0.003 |
| Insufficient | 121(19.4) | 503(80.6) |  |  |
| Sufficient | 429(25.3) | 1264(74.7) |  |  |
| **Peer-influenced purchase of SSBs** |  |  | 2.908 | 0.088 |
| No | 202(21.9) | 721(78.1) |  |  |
| Yes | 348(25.0) | 1046(75.0) |  |  |
| **Peer-sharing of SSBs** |  |  | 15.994 | *p*＜0.001 |
| No | 127(18.3) | 566(81.7) |  |  |
| Yes | 423(26.0) | 1201(74.0) |  |  |
| **Parents’ attitudes toward SSBs** |  |  | 1.492 | 0.474 |
| Supportive | 69(26.5) | 191(73.5) |  |  |
| Non-supportive | 283(23.8) | 908(76.2) |  |  |
| Indifferent | 198(22.9) | 668(77.1) |  |  |
| **Household availability of SSBs** |  |  | 0.174 | 0.677 |
| No | 196(23.3) | 647(76.7) |  |  |
| Yes | 354(24.0) | 1120(76.0) |  |  |

**Supplementary Table 7.** Relationships between health-related behaviors, peer-related factors, home-related factors and beverages of special uses consumption

| **Variables** | **Beverages for special uses Consumption(n=2317)** | | | |
| --- | --- | --- | --- | --- |
|  | **Yes**  **(n=702)** | **No**  **(n=1615)** | **χ^2^/t** | ***p*-Value** |
| **Daily water intake** |  |  | 27.118 | *p*＜0.001 |
| ＜1000 ml/day | 140(25.2) | 415(74.8) |  |  |
| 1000~1500 ml/day | 206(26.6) | 568(73.4) |  |  |
| ＞1500 ml/day | 356(36.0) | 632(64.0) |  |  |
| **Outdoor activity time** |  |  | 34.495 | *p*＜0.001 |
| ＜60 minutes/day | 266(24.4) | 826(75.6) |  |  |
| ≥60 minutes/day | 436(35.6) | 789(64.4) |  |  |
| **Weekday sleep time** |  |  | 1.303 | 0.254 |
| Insufficient | 524(29.7) | 1241(70.3) |  |  |
| Sufficient | 178(32.2) | 374(67.8) |  |  |
| **Weekend sleep time** |  |  | 6.521 | 0.011 |
| Insufficient | 164(26.3) | 460(73.7) |  |  |
| Sufficient | 538(31.8) | 1155(68.2) |  |  |
| **Peer-influenced purchase of SSBs** |  |  | 5.181 | 0.023 |
| No | 255(27.6) | 668(72.4) |  |  |
| Yes | 447(32.1) | 947(67.9) |  |  |
| **Peer-sharing of SSBs** |  |  | 5.141 | 0.023 |
| No | 187(27.0) | 506(73.0) |  |  |
| Yes | 515(31.7) | 1109(68.3) |  |  |
| **Parents’ attitudes toward SSBs** |  |  | 3.597 | 0.166 |
| Supportive | 72(27.7) | 188(72.3) |  |  |
| Non-supportive | 348(29.2) | 843(70.8) |  |  |
| Indifferent | 282(32.6) | 584(67.4) |  |  |
| **Household availability of SSBs** |  |  | 4.839 | 0.028 |
| No | 232(27.5) | 611(72.5) |  |  |
| Yes | 470(31.9) | 1004(68.1) |  |  |
